# Constant light in early life induces fear-related behavior in chickens with suppressed melatonin secretion and disrupted hippocampal expression of clock- and BDNF-associated genes

**DOI:** 10.1186/s40104-022-00720-4

**Published:** 2022-06-21

**Authors:** Yang Yang, Wei Cong, Jie Liu, Mindie Zhao, Peirong Xu, Wanwan Han, Deyun Wang, Ruqian Zhao

**Affiliations:** 1 MOE Joint International Research Laboratory of Animal Health & Food Safety, Institute of Immunology, Nanjing Agricultural University, Nanjing, 210095 People’s Republic of China; 2 Key Laboratory of Animal Physiology & Biochemistry, College of Veterinary Medicine, Nanjing Agricultural University, Nanjing, 210095 People’s Republic of China; 3 Institute of Traditional Chinese Veterinary Medicine, College of Veterinary Medicine, Nanjing Agricultural University, Nanjing, 210095 People’s Republic of China

**Keywords:** BDNF/ERK, Constant light, Fear-related behavior, Hippocampus

## Abstract

**Background:**

Light management plays an important role in the growth and behavior of broiler chickens. Constant light in early post hatch stage has been a common practice in broiler industry for improving growth performance, while whether and how constant light in early life affects the behavior of broiler chickens is rarely reported.

**Results:**

In this study, newly hatched chicks were kept in either constant (24 L:0 D, LL) or (12 L:12 D, LD) photoperiod for 7 d and then maintained in 12 L:12 D thereafter until 21 days of age. Constant light increased the average daily feed intake but not the body weight, which led to higher feed conversion ratio. Chickens in LL group exhibited fear-related behaviors, which was associated with higher corticosterone, lower melatonin and 5-HT levels. Concurrently, constant light exposure increased the mRNA expression of clock-related genes and suppressed the expression of antioxidative genes in the hippocampus. Moreover, brain derived neurotrophic factor/extracellular signal-regulated kinase (BDNF/ERK) pathway was suppressed in the hippocampus of chickens exposed to constant light in the first week post hatching.

**Conclusions:**

These findings indicate that constant light exposure in early life suppress melatonin secretion and disrupts hippocampal expression of genes involved in circadian clock and BDNF/ERK pathway, thereby contributing to fear-related behaviors in the chicken.

**Supplementary Information:**

The online version contains supplementary material available at 10.1186/s40104-022-00720-4.

## Introduction

Light is an important factor affecting the growth and development, behavior and welfare, as well as stress and immune responses [1, 2]. Photoperiod, light wavelength and intensity are three major variables in light management that affect growth and behavior of broiler chickens [3, 4]. Broiler chickens are usually maintained under lighting regimes (hours in light = L, dark = D) with constant (24 L:0 D) or near-constant photoperiod (23 L:1 D) in modern industrialized farms, especially during the early post hatch period, because it was demonstrated that constant lighting regimen stimulates feed intake and promote growth [5]. However, there are some controversial reports questioning the long photoperiods application in broiler industry. It was reported [6] that 12 L:12 D photoperiod improves meat quality attributes in Ross 308 broilers chickens, compared with long photoperiod (20 L: 4 D). Also, near-constant photoperiod (23 L:1 D) increases stress and fear levels in Ross 308 broilers chickens [7], disrupts circadian clock gene expression and cecal microbiome diversity in Hy-Line Brown Layer chickens [8]. Nevertheless, the mechanisms underlying constant or near-constant light-induced malfunctions in the chicken remain largely unknown.

In mammals, constant light has been reported to induce circadian disruption, fatigue, irritability, depression and anxiety [9, 10]. In searching for the underlying mechanisms, many studies focus on melatonin, a hormone secreted by the pineal gland in response to the external light cues [11]. Constant light disrupts circadian rhythm through inhibiting melatonin secretion from the pineal gland. Previously, we found that mice exposed to constant light exhibited fear-related behavior with lower melatonin and higher corticosterone (CORT) levels in the blood [12]. Higher plasma CORT levels are linked with depression-like behavior in weanling mice [13] and adult rats [14] exposed to dim light at night. CORT is commonly used as an important indicator of stress in both rodents and chickens [15]. Conversely, melatonin and its precursor 5-hydroxytryptamine (5-HT) are related to improved behavior and wellbeing in both mammalian [16] and avian [17] species. Melatonin is reported to play important roles in regulating circadian clock, alleviating depression and anxiety, and mitigating oxidation-induced injuries [18, 19].

Hippocampus is the key brain structure governing many important functions including learning and memory, as well as depressive and anxiety-like behaviors [20]. Brain derived neurotrophic factor (BDNF) is critical for the hippocampal neurogenesis and synaptic plasticity [21, 22]. BDNF acts through binding to its receptor tropomyosin receptor kinase B (TrκB), thereby triggering the activation of phosphatidylinositol 3-kinase (PI3K) and/or extracellular signal-regulated kinase (ERK) pathways [23]. Previously, we reported that mice exposed to constant light for 3 weeks show reduced hippocampal neurogenesis and impaired cognitive behaviors, which is associated with suppressed BDNF/TrκB/ERK pathway in hippocampus [24]. In birds, hippocampus performs the same functions as in mammals, including spatial memory [25] and fear-related behaviors [4]. Chronic CORT exposure disrupted hippocampal expression of clock genes in the chicken [26]. Moreover, light exposure has been reported to influence the expression of clock genes in the pineal gland, hypothalamus and retina of chicks [27, 28]. However, no data is available regarding the effects of constant light exposure on hippocampal expression of BDNF/TrκB/ERK and clock genes in the chicken.

Therefore, in this study we exposed the chicks to either constant (24 L:0 D) or 12 L:12 D photoperiods for 1 week after hatching, to elaborate the effects of constant light in early life on fear-related behaviors, serum 5-HT, melatonin and CORT levels, and the hippocampal expression of genes involved in BDNF/ERK pathway and circadian clocks in the chicken.

## Materials and methods

### Ethics statement

The experimental protocol was approved by the Animal Ethics Committee of Nanjing Agricultural University. The project number is 31972638. The sampling procedures according to the “Guidelines on Ethical Treatment of Experimental Animals” (2006) No.398 set by the Ministry of Science and Technology, China.

### Animals and experimental design

The chicken model employed in this study is a locally bred chicken line used for meat production in China. Eighty 1-day-old male Yellow-footed chickens were purchased from Changzhou Lihua Livestock and Poultry Co., Ltd., and randomly divided into normal (LD) and constant (LL) photoperiods for the 1st week after hatching. Light regime in LD group was 12 h light: 12 h dark, with light on at 07:00 h and off at 19:00 h; light regime in LL group was 24 h light:0 h dark. The light intensity was about 200 lx for both groups in the first week, due to the illumination from the heat lamps. From 8 d to 21 d, chickens from both groups were maintained under the same light regime (12 L:12 D) until the end of the experiment (21 d). The light intensity was about 80 lx for both groups from the second week when the heat lamps were removed. The birds in LD and LL groups were housed in 2 adjacent rooms with the same condition except the lighting regime set for each group. Birds in each room were housed in 2 individual cages with 20 birds per cage for the 1st week, and then distributed into 4 cages with 10 birds per cage thereafter till the end of the experiment. Feed and water were provided ad libitum. The room temperature was set according to the standard established by the breeding company. In detail, the temperature was 35 °C for the first 2 d, 32–34 °C from 3 to 7 d, 30–32 °C from 8 to 14 d, and 27–30 °C from 15 to 21 d. The feed consumption per cage was recorded daily, and body weight was recorded every week. By the end of the experiment, chickens were anesthetized with sodium pentobarbital and the brain was quickly separated from the skull. The brain was separated in the middle and both hemispheres of the rostral and caudal hippocampus were dissected as described in a previous publication [29] according to the chicken brain atlas [30]. Both hemispheres were combined and frozen immediately in liquid nitrogen and stored at − 80 °C until use. The tissue was homogenized, and part of the homogenate was used for mRNA or protein extraction. All chickens were killed by rapid decapitation that is considered acceptable for euthanasia of birds according to American Veterinary Medical Association (AVMA) Guidelines for the Euthanasia of Animals: 2013 Edition.

### Behavior tests

On 7 days of age, chickens from both groups were captured from their home pen and carried individually to the adjacent room where the behavior tests were performed. Open field test, balance beam test, and the tonic immobility test were carried out to explore the effects of constant light on fear-related behaviors in chickens.

#### Open field test

The chicken to be tested was placed in the center of a 58 cm × 58 cm open field box with sides 70 cm high. This box was made of white wood and the floor was marked off into 25 squares of 12 cm × 12 cm each, illuminated by a 100 W overhead bulb [31]. After the test for each chicken, the floor of the box was cleaned with towels wetted with 70% ethanol. The behavioral performances were filmed with the observer standing out of the view of the tested chicken. The recorded videos were then analyzed by a fellow student who was not aware of the experimental design. The time of the first step, the counts of grid crossed, defecation, steps, escape attempts and vocalizations with 10 min of observation were recorded for statistical analysis.

#### Balance beam test

The test was conducted using an elevated narrow balance beam (6 cm wide and 35 cm long). The balance beam was 22 cm high, so that chicks could jump down onto soft bedding without injuring themselves. The balance beam was used to assess fear-related behaviors, similar to the test in rodents [32]. The test was performed as follows. Each chick was placed on the starting line at one end of the balance beam and then allowed to walk. We recorded the following parameters: (i) the time of the chick stayed on the beam and (ii) the distance the chick walked on the beam. If a chick stayed on the starting line or was afraid of walking on the beam, it received a score of 0 and scored as 120 s.

#### Tonic immobility test

Chicks were placed on their backs in a metal cradle and restrained for 10 s to induce tonic immobility (TI) by the experimenter placing one hand on the bird’s chest and another over its head with the head hanging down. Chickens that did not remain still for 10 s were allowed maximum 5 times to induce TI. TI was successfully induced for all the chickens used in this study in less than 5 times, and the exact duration of the successful TI was recorded. When the chicken remained still for over 6 min, the TI test was terminated and the TI score for this chicken was 6 min.

### Measurement of corticosterone, melatonin and 5-hydroxytryptamine

On 7 and 21 days of age, chickens were randomly selected from each group for sampling. Chickens were sacrificed and blood samples were collected. Serum corticosterone (CORT) concentration was determined by Enzyme Immunoassay (EIA) kit (No. ADI-900-097, Enzo, Farmingdale, NY, USA) following the manufacturer’s instructions. Serum melatonin levels were measured using Chicken Melatonin (MT) ELISA Kit (MM-34278O1, ImmunoWay Biotechnology, USA) following the manufacturer’s instructions. Serum 5-hydroxytryptamine (5-HT) levels were measured using Chicken 5-HT ELISA Kit (E-EL-0033c, Elabscience, USA) following the manufacturer’s instructions.

### RNA isolation and real-time PCR

High quality total RNA was isolated from 30 mg hippocampal tissue using 600 μL Trizol reagents (Invitrogen, Carlsbad, California, USA). One microgram RNA was reverse-transcribed according to the manufacturer’s protocol (Vazyme Biotech, Nanjing, Jiangsu, China). Four microliter cDNA was diluted (1:25) and then used for real-time PCR in a QuantStudioTM 6 Flex Real-Time PCR System (Applied Biosystems, Foster City, California, USA). Peptidylprolyl isomerase A (PPIA) was used as an internal control to normalize the technical variations. Data were analyzed using the method of 2^-ΔΔCT^ and presented relative to the CON group. All primers (Table 1) were synthesized by Suzhou GENEWIZ Biological Technology Co., Ltd. (Suzhou, Jiangsu, China).

**Table 1:** The primers sequences for RT-PCR

Target genes	Primer sequences (5’ to 3′)	
*clock*	F: GATCACAGGGCACCTCCAATA	R: CTAGTTCTCGCCGCCTTTCT
*baml1*	F: GTAGACCAGAGGGCGACAG	R: ATGAAACTGAACCAGCGACTC
*cry1*	F: GATGTGGCTATCCTGTAGTTCCT	R: GCTGCTGGTAGATTTGTTTCAT
*cry2*	F: GCACGGCTGGATAAACACT	R: AAATAAGCGGCAGGACAAA
*per2*	F: ATGAAACGAGCCATCCCG	R: CAGTTGTCGTGATTTTGCCTA
*per3*	F: CAGTGCCTTTGTTGGGTTAC	R: GATGGATTCACAAAACTGGAC
*rorα*	F: GGGGATGTCTCGAGATGCTG	R: TGCTTTGCTACCTTCAGGGG
*rev-erbα*	F: CAGCGGTTTCCAGTCATCCT	R: TCACTCTTTGGTGCCCCATC
*5-hta*	F: AGAACACGGAGGCCAAGC	R: ACGGCAACCAGCAGAGGA
*5-htb*	F: CACGGACCACGTCCTCTACAC	R: TTTCTTTGGCGTCTGCTTCA
*maoa*	F: ATTCCTCCTGAAGCACCAT	R: CACTGCCTCACATACCAC
*maob*	F: AGGCTGGAGAAAAAGCAGCA	R: CCCGGTACAGATGGCAAGTT
*nrf2*	F: GGCCACCCTAAAGCTCCATT	R: GGCTTCACTGAACTGCTCCT
*keap1*	F: TCAACTGGGTGCAGTACGAC	R: TCTGCGCCAGGTAATCCTTG
*sod1*	F: GAGCGGGCCAGTAAAGGTTA	R: CCCTTTGCAGTCACATTGCC
*sod2*	F: TACAGCTCAGGTGTCGCTTC	R: GCGAAGGAACCAAAGTCACG
*nqo1*	F: CGCACCCTGAGAAAACCTCT	R: AAGCACTCGGGGTTCTTGAG
*cat*	F: GCGCCCCGAACTATTATCCA	R: ATACGTGCGCCATAGTCAGG
*gctc*	F: GGACGCTATGGGGTTTGGAA	R: AGGCCATCACAATGGGACAG
*gpx1*	F: CTGCAACCAATTCGGGCAC	R: CACCTCGCACTTCTCGAACA
*gpx2*	F: CGCCAAGTCCTTCTACGACC	R: GGTGTAATCCCTCACCGTGG
*gpx3*	F: CGAAAGTACGCGGGGAAGAT	R: GGACGACAAGTCCATAGGGC
*bdnf*	F: GACATGGCAGCTTGGCTTAC	R: GTTTTCCTCACTGGGCTGGA
*trκb*	F: TGACTGTGGTGGATTCAGGC	R: TGGCGAAAAGGCTTCTTGGA
*dcx*	F: GCAGCTGCCACAGGTAGTAA	R: ACTGCTTGGATTTGGGCGTA
*ppia*	F: TTACGGGGAGAAGTTTGCCG	R: TGGTGATCTGCTTGCTCGTC

### Total protein extraction and Western blotting

Total protein was extracted from about 50 mg frozen hippocampus samples. Protein concentrations were measured using BCA Protein Assay kit (NO.23227, Thermos Scientific, Rockford, Illinois, USA) according to the manufacturer’s instructions. Protein (50 μg/lane) was loaded for electrophoresis on a 6–14% SDS-PAGE gel and transferred onto a nitrocellulose membrane. After transfer, the membranes were blocked with 4% milk and then incubated with primary and secondary antibodies. Western blot analysis for NRF2 (16396–1-AP, proteintech, USA, diluted 1:1000), BDNF (ab108319, Abcam, USA, diluted 1:500), TrκB (bs-0288R, Bioss, USA, diluted 1:1000), Doublecortin (DCX; 4604, Cell Signaling Technology, USA, diluted 1:1000), ERK (4695, Cell Signaling Technology, USA, diluted 1:1000), phospho-ERK (4370, Cell Signaling Technology, USA, diluted 1:1000) was performed, using tubulin α (BS1699, Bioworld, diluted 1:10,000) as loading control. Images were captured by VersaDoc 4000MP system (Bio-Rad, USA) and the band density was analyzed with Quantity One software (Bio-Rad, USA).

### Statistical analysis

All data are presented as means ± SEM. The behavior data were analyzed using U-Test, and the hormones and gene expression data were analyzed by T-Test for independent samples with SPSS 20.0. The differences were considered statistically significant when *P* < 0.05.

## Results

### Effect of constant light in early life on feed conversion ratio, serum CORT, melatonin and 5-HT concentration

**Fig. 1:**
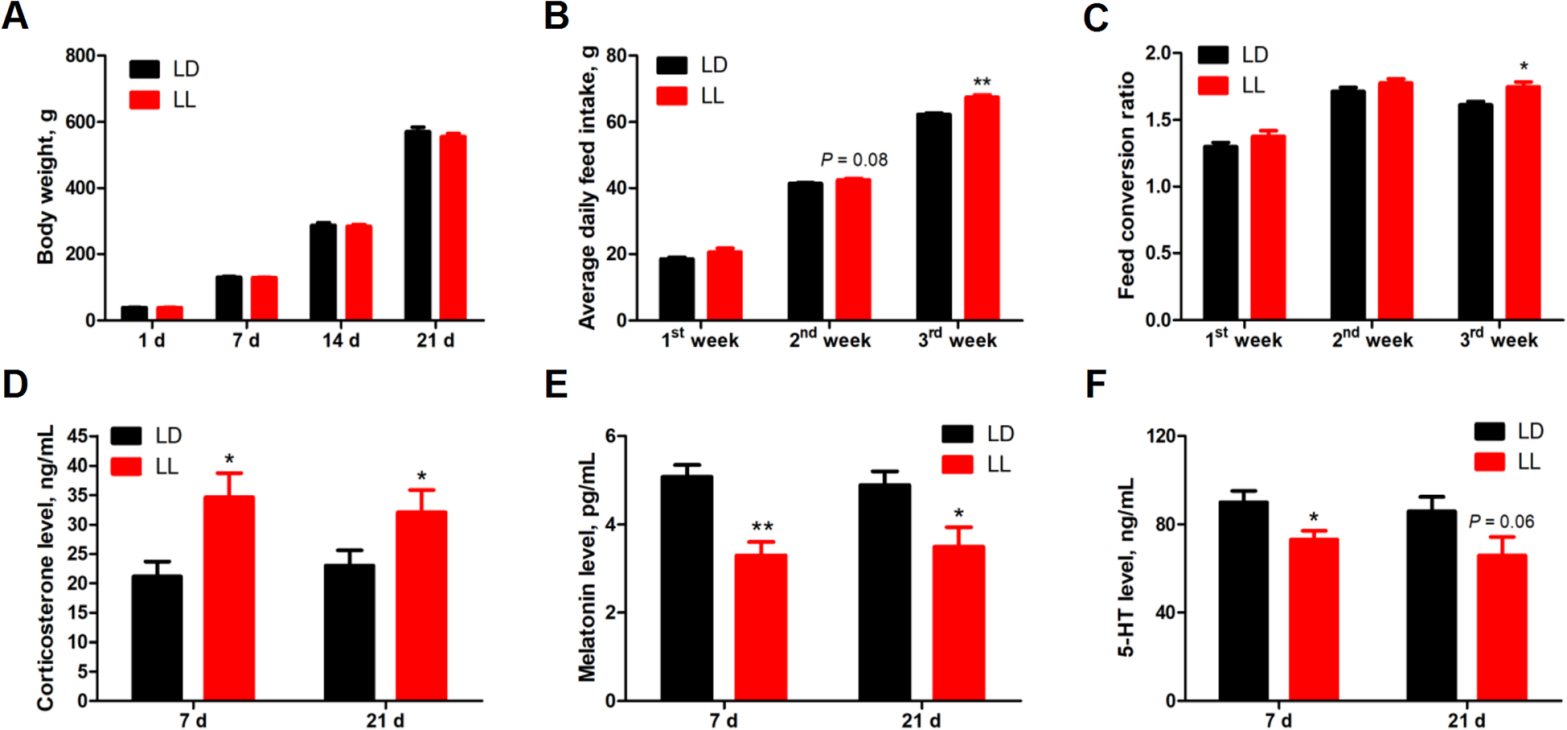
Effect of constant light exposure on growth performance and serum hormone concentration. **A** Body weight (1 d, 7 d, *n* = 39 (LD), *n* = 40 (LL); 14 d, 21 d, *n* = 20); **B** Average daily feed intake (1st week, *n* = 2; 2nd and 3rd week, *n* = 4); (**C**) Feed conversion ratio (1st week, *n* = 2; 2nd and 3rd week, *n* = 4); **D** Serum corticosterone content (*n* = 10); **E** Serum melatonin content (*n* = 10); **F** Serum 5-HT content (*n* = 10). Values are mean ± SEM, *P < 0.05, **P < 0.01, compared with LD

Constant light did not affect (*P* > 0.05) the body weight (Fig. 1A), but increased the average daily feed intake in 2nd week (*P* = 0.08) and 3rd week (*P* < 0.05) (Fig. 1B), which led to significantly higher feed conversion ratio (*P* < 0.05) in 3rd week (Fig. 1C). Meanwhile, serum CORT levels (Fig. 1D) were significantly higher (*P* < 0.05), while serum melatonin (Fig. 1E) levels were significantly lower (*P* < 0.05) in LL group at both 7 and 21 days of age. Chickens from LL group exhibited significantly lower (*P* < 0.05) serum 5-HT concentration (Fig. 1F) at 7 days of age, with a trend of decrease (*P* = 0.06) detected at 21 days of age, as compared with their counterparts from LD group.

### Effect of constant light in early life on the fear-related behaviors in chickens

**Fig. 2:**
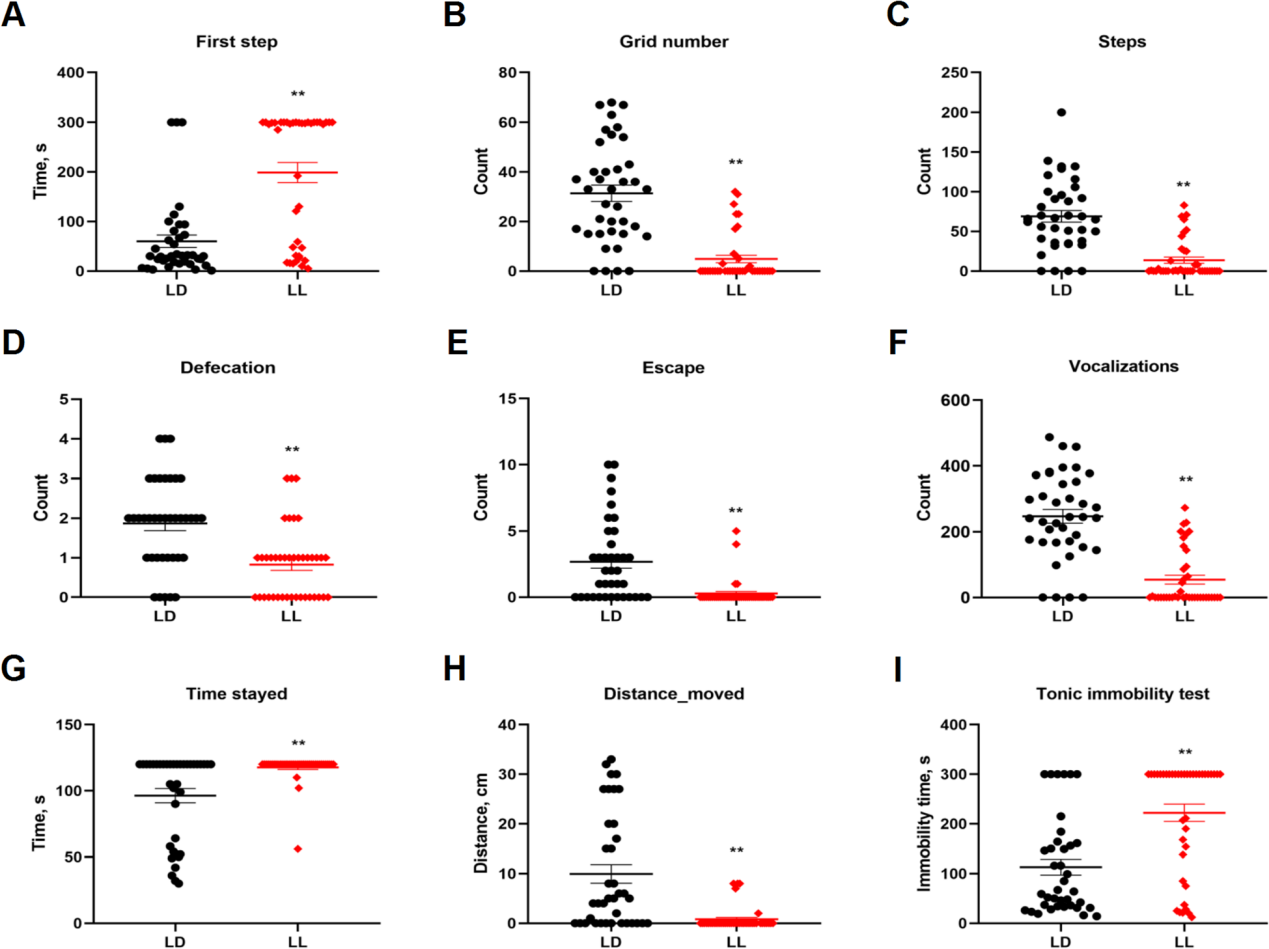
Effect of constant light exposure on anxiety and fear-related behavior. To elaborate the effect of constant light exposure on locomotor activity and fear-related activity, the open-field test, balance beam test and tonic immobility test were performed. **A** The times of first step; **B-F** The counts of grid number, defecation, steps, escape and vocalizations in the open field test; **G** Times stayed in balance beam test; **H** The distance moved in balance beam test; **I** The immobility time in tonic immobility test. Values are mean ± SEM (LD, *n* = 39, LL, *n* = 40); **P < 0.01, compared with LD. The data were analyzed using U-Test with SPSS 20.0

Chickens from LL group spent significantly longer (*P* < 0.01) time before taking the first step in the open filed test (Fig. 2A). Moreover, the counts of the grid crossed (Fig. 2B) and the counts of steps (Fig. 2C), defecation (Fig. 2D), escape (Fig. 2E) and vocalizations (Fig. 2F) were all significantly decreased (*P* < 0.01) in LL group. Also, the time stayed on the balance beam (Fig. 2G) was significantly increased (*P* < 0.01), and the distance moved along the beam (Fig. 2H) was significantly decreased (*P* < 0.01) in LL group. In tonic immobility test, chickens from LL group showed significantly longer (*P* < 0.01) immobility time, compared to their LD counterparts (Fig. 2I). See Table 2 for detailed behavioral data. Raw data of behavioral test were showed in Supplementary Table 1. These behavior results indicate that constant light in early life significantly increased fear-related behaviors in chickens.

**Table 2:** Effect of lighting time on the behavior of Yellow-footed chickens

	LD	LL	*P*-value	T-value	df
**Open field test**					
First step time, s	60.11 ± 12.71	198.80 ± 12.72	0.00	−5.80	65.18
Grid number, n	31.37 ± 3.30	4.88 ± 1.51	0.00	7.30	51.96
Defacation, n	69.05 ± 7.25	13.70 ± 3.83	0.00	6.75	56.35
Steps, n	1.87 ± 0.82	0.83 ± 0.14	0.00	4.52	71.10
Escape, n	2.68 ± 0.49	0.28 ± 0.16	0.00	4.67	44.82
Tweet, n	247.26 ± 21.07	54.40 ± 13.41	0.00	7.72	63.22
**Balance test**					
Time_stayed, s	96.32 ± 5.35	117.70 ± 1.66	0.00	−3.81	44.08
Distance _moved, cm	9.95 ± 1.87	0.83 ± 0.37	0.00	4.77	39.94
**Tonic immobility test**					
Immobility time, s	113.08 ± 15.88	222.32 ± 17.62	0.00	−4.61	75.54

Data are presented as means ± SEM; LD, *n* = 39; LL, *n* = 40

Raw data of behavioral test were showed in Additional file 1

### Effect of constant light in early life on hippocampal 5-HT receptors and clock genes mRNA expression in chickens

**Fig. 3:**
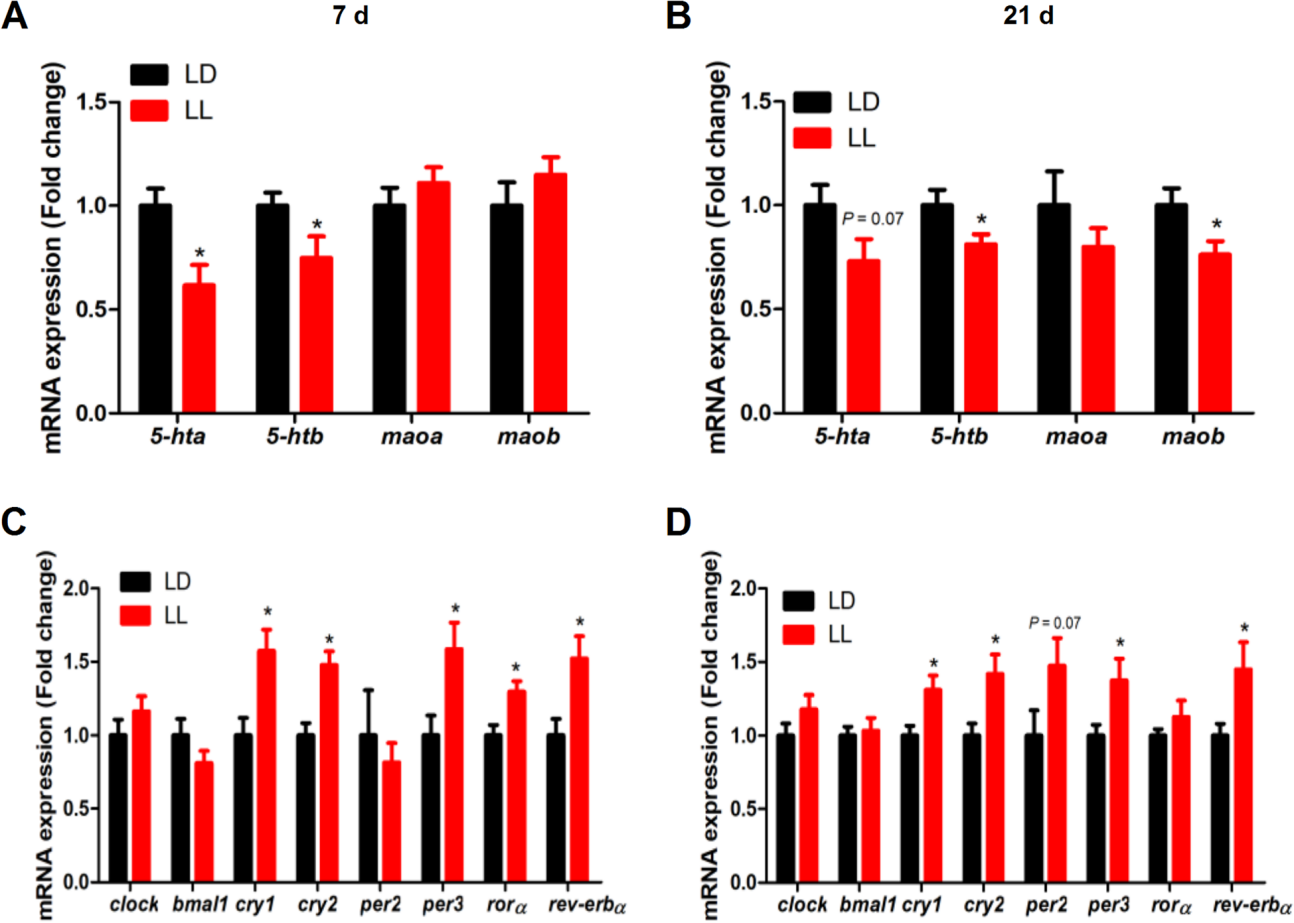
Effect of constant light exposure on 5-HT receptor and clock related gene mRNA expression in chicken’s hippocampus. **A-B** The mRNA expression of *5-hta*, *5-htb*, *maoa* and *maob* in 7 d and 21 d chickens; **C-D** The circadian clock genes mRNA expression in 7 d and 21 d chickens. Values are mean ± SEM (*n* = 12); *P < 0.05, compared with LD

Hippocampal expression of 5-HT receptors *5-hta* and *5-htb* mRNA was significantly decreased (*P* < 0.05) in LL chickens at 7 days of age (Fig. 3A). At 21 days of age, chickens from LL group showed a significant down-regulation (*P* < 0.05) of hippocampal expression of *5-htb* and *maob*, the key enzymes for 5-HT degradation (Fig. 3B), with a tendency of down-regulation (*P* = 0.07) for *5-hta* mRNA. In addition, hippocampal mRNA expression of clock-related genes, *cry1*, *cry2*, *per3*, *rorα* and *rev-erbα*, was significantly increased (*P* < 0.05) in LL group at 7 days of age (Fig. 3C). At 21 days of age, *cry1*, *cry2*, *per3* and *rev-erbα* mRNA expression was significantly increased (*P* < 0.05), while *per2* mRNA expression tended (*P* = 0.07) to increase (Fig. 3D) in the hippocampus of LL chickens.

### Effect of constant light in early life on oxidative stress related genes mRNA and protein expression chicken hippocampus

**Fig. 4:**
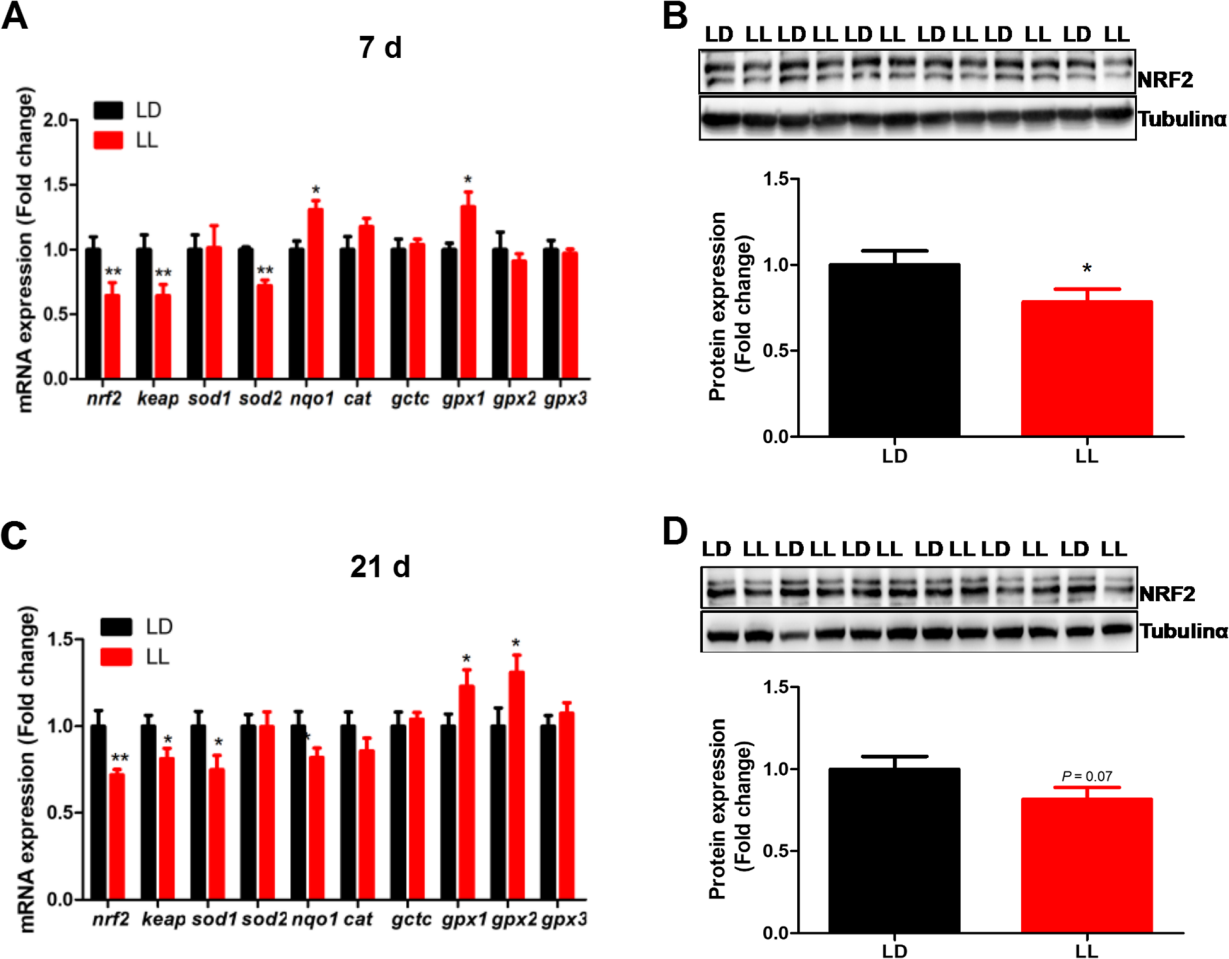
Effect of constant light exposure on oxidative stress related gene mRNA and. Protein expression in chicken’s hippocampus. **A** Oxidative stress related gene mRNA expression in 7 d chickens (*n* = 12); **B** Nrf2 protein expression in 7 d chickens (*n* = 6); **C** Oxidative stress related gene mRNA expression in 7 d chickens (*n* = 12); **D** Nrf2 protein expression in 7 d chickens (*n* = 6). Values are mean ± SEM; *P < 0.05, compared with LD

Hippocampal expression of oxidative stress-related genes was modified by constant light exposure. The expression of NF-E2-related factor 2 (*nrf2*), Kelch-like ECH-associated protein 1 (*keap1*) and superoxide dismutase 2 (*sod2*) were significantly decreased (*P* < 0.05), while that of *nqo1* and *gpx1* was significantly increased (*P* < 0.01) in LL group at 7 days of age (Fig. 4A). Accordantly, NRF2 protein content in hippocampus was significantly decreased (*P* < 0.05) in 7-day-old LL chickens (Fig. 4B). Similar changes in the pattern of oxidative stress-related genes were detected at 21 d, with decreased *nrf2*, *keap*, *sod1* and *nqo1* (*P* < 0.05) and increased (*P* < 0.05) *gpx1* and *gpx2* mRNA expression (Fig. 4C) in the hippocampus of LL chickens. NRF2 protein content tended to decrease (*P* = 0.07) in the hippocampus of LL chickens at 21 days of age (Fig. 4D).

### Effect of constant light in early life on BDNF/TrκB/ERK mRNA and protein expression in chicken hippocampus

**Fig. 5:**
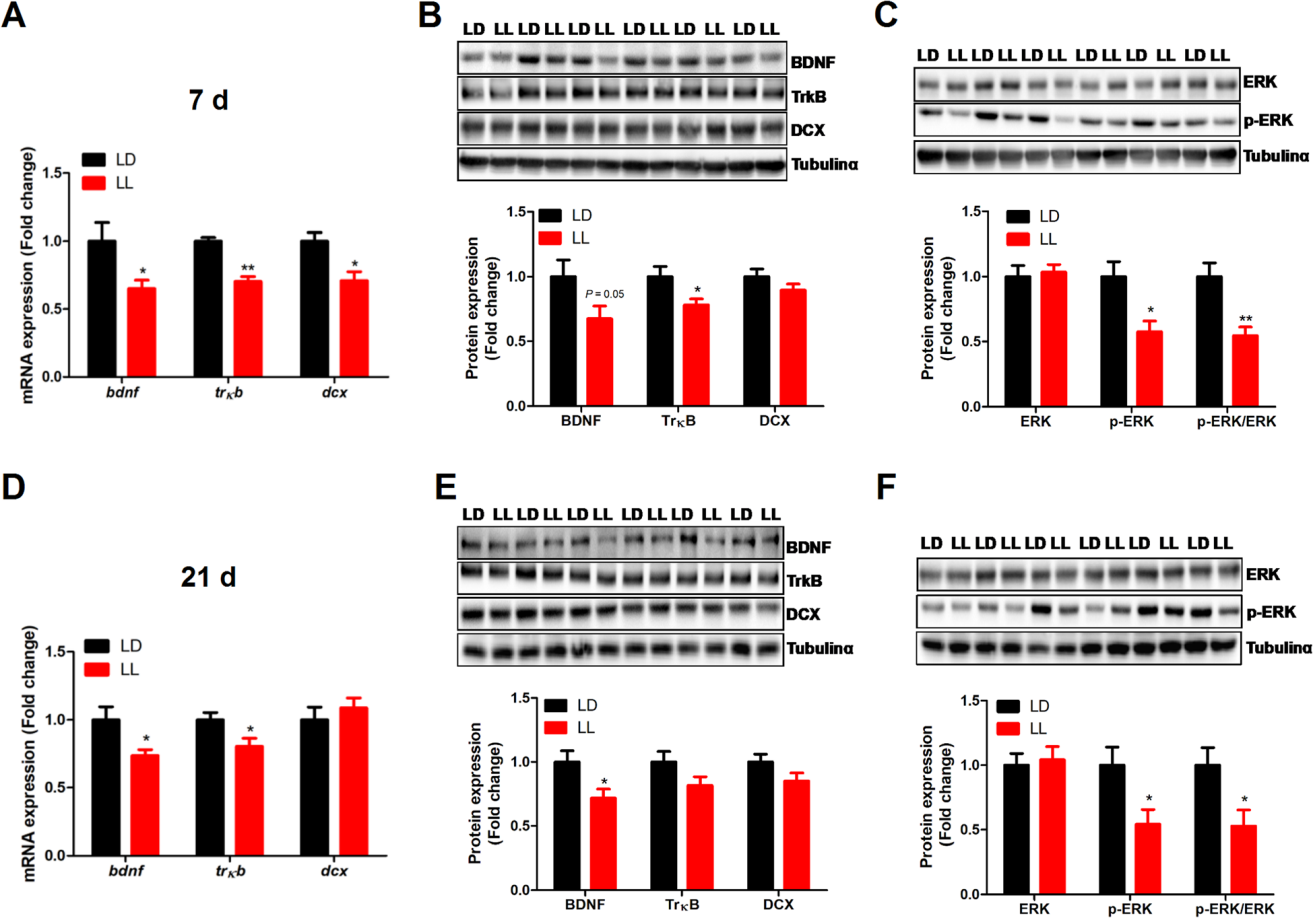
Effect of constant light exposure on BDNF/TrκB/ERK pathway mRNA and protein expression in chicken’s hippocampus. **A** The mRNA expression of *bdnf*, *trκb* and *dcx* in 7 d chickens (*n* = 12); **B** BDNF, TrκB and DCX protein expression in 7 d chickens (*n* = 6); **C** ERK and p-ERK protein expression in 7 d chickens (*n* = 6); **D** The mRNA expression of *bdnf*, *trκb* and *dcx* in 21 d chickens (*n* = 12); **E** BDNF, TrκB and DCX protein expression in 21 d chickens (*n* = 6); **F** ERK and p-ERK protein expression in 21 d chickens (*n* = 6). Values are mean ± SEM; *P < 0.05, **P < 0.01, compared with LD

Constant light exposure significantly decreased (*P* < 0.05) hippocampal expression of *bdnf*, *trκb* and *dcx* mRNA (Fig. 5A). Accordantly, TrκB and p-ERK protein content was significantly decreased (*P* < 0.05), while BDNF protein tended to decrease (*P* = 0.05), in the hippocampus of LL chickens at 7 days of age (Fig. 5B and C). Similar alterations occur at 21 d, with significantly decreased (*P* < 0.05) *bdnf* and *trκb* mRNA (Fig. 5D) and BDNF and p-ERK protein (Fig. 5E and F) determined in the hippocampus of LL chickens.

## Discussion

In this study, the light intensity (200 lx in the 1st week and about 80 lx thereafter) was much brighter than the commercial conditions (less than 30 lx). A study performed under commercial light intensity (29 ± 2.3 lx) reported that 24 h constant light increased food intake but not body weight in 21 d males Cobb 500 and Ross 308 broiler chickens [5]. Despite the difference in light intensity and the chicken lines, we also found that constant light regime increased feed intake but did not increase the body weight gain, which led to higher FCR value. Therefore, the constant light regime does not provide benefits either to chickens or farmers.

In the present study, chickens show unique behaver patterns in the open field test. Most of the chickens from LL group did not move within 6 min, so the number of grids crossed and attempts of escape were basically zero. In addition, the number of defecation and vocalizations was also significantly reduced in LL group. This finding agrees with a previous publication that increased defecation latency and decreased defecation frequency were associated with anxiety-like behavior in neonatal broiler chicks [33]. Also, chicks with higher plasma corticosterone levels show longer vocalization latency [34]. Defection and vocalization are normal physiological responses for a chicken when separated from the group in the open field test. Decreased frequency of defection and vocalization in LL group indicates fear-related condition. Similar fear-related responses were observed in the balance beam test, chickens from LL group showing longer latency, shorter walking distance and longer immobility time. Our findings in chickens are in line with a previous report that near-constant light (23 L:1 D) exposure increased stress and fear-related behaviors in Ross 308 broilers chickens [7].

Melatonin is a potent antioxidant by scavenging reactive oxygen species [35]. Constant light-induced depressive-like behavior in rodents is usually associated with low melatonin and high oxidative stress levels [36, 37]. Indeed, we detected lower plasma melatonin and 5-HT levels in LL chickens, which was associated with suppressed hippocampal mRNA expression of 5-HT receptors at both 7 and 21 days of age. Similar results have been reported that 23 L:1 D photoperiod significantly reduced plasma melatonin in 7-week-old broiler chickens, as compared with 12 L:12 D photoperiod [38]. In addition, we found that constant light exposure increased the plasma CORT levels in the chicken at both 7 and 21 days of age. CORT levels are associated to depression-like behavior in rodents [21]. The same is true for chicken that increased fear-related behavior is related to lower serum levels of melatonin and 5-HT, and higher serum levels of CORT.

Nrf2 and its endogenous inhibitor, Keap1, play critical roles in counteracting oxidative stress [39]. In this study, constant light exposure reduced *nrf2/keap1* and *sod* mRNA expression, as well as Nrf2 protein content in chicken hippocampus at both 7 and 21 days of age. In agreement with our findings, constant light was reported to induce oxidative stress in rat hippocampus, cortex and cerebellum [40] and melatonin was reported to rescue the suppressed Nrf2 expression in the thymus of rats exposed to constant light [41].

A number of studies indicate that constant light exposure influences the mRNA expression of clock-related genes, including *clock*, *bmal1*, *cry*, and *per* [42, 43]. Disrupted expression of clock-related genes is associated with impaired hippocampal neurogenesis and depressive-like behavior in mice [44, 45]. Similarly, we also found that constant light exposure significantly increased the mRNA expression of clock-related genes, including *cry1*, *cry2* and *rev-erbα,* in the hippocampus of chickens at both 7 and 21 days of age.

BDNF/ERK pathway, which is critical for hippocampal neurogenesis [21], plays an important role in regulating depressive-like behavior [20]. In the present study, we found that constant light exposure significantly suppressed BDNF/ERK pathway in the hippocampus of chickens at both 7 and 21 days of age. These results are consistent with our previous findings in mice that constant light suppressed BDNF/ERK pathway in hippocampus [24]. Melatonin was able to rescue chronic restraint stress-induced depressive-like behavior in mice via activating the hippocampal BDNF/TrκB signaling pathway [46]. The role of hippocampal BDNF in the chicken is less understood, yet in a chick anxiety-depression model, hippocampal BDNF response was linked to stress resilience [47].

## Conclusion

In conclusion, our study shows that constant light exposure in early life induces fear-related behaviors in the chicken, which is accompanied by higher CORT and lower melatonin/5-HT levels in the blood. Constant light exposure in early life disrupted expression pattern of genes involved in circadian clock, oxidation stress, and BDNF/TrκB/ERK pathway in chicken hippocampus. These findings extend our understanding on hormonal and hippocampal responses to constant light exposure in the chicken, and provide implications for the light management in broiler industry in favor of production efficiency as well as chicken behavior and welfare.

## Supplementary Information

**Additional file 1.**

## Data Availability

Not applicable.

## Abbreviations

5-HT: 5-hydroxytryptamine
ADFI: Average daily feed intake
BDNF: Brain derived neurotrophic factor
CORT: Corticosterone
EIA: Enzyme Immunoassay
ERK: Extracellular signal-regulated kinase
FCR: Feed conversion ratio
Keap1: Kelch-like ECH-associated protein 1
LD: Natural photoperiod
LL: Constant light
MT: Melatonin
Nrf2: NF-E2-related factor 2
PPIA: Peptidylprolyl isomerase A
PI3K: Phosphatidylinositol 3-kinase
SOD: Superoxide dismutase
TrκB: Tropomyosin receptor kinase B
